# Age-related differences in the presentation, management, and outcomes of lower gastrointestinal bleeding: a retrospective multinational cohort study

**DOI:** 10.1016/j.lanepe.2026.101775

**Published:** 2026-07-09

**Authors:** Francisco Vara-Luiz, Carolina Palma, Paulo Mascarenhas, Tony C. Tham, Marianna Arvanitakis, Enrique Rodriguez-de-Santiago, Isabel Pedroto, Diogo Simas, Franco Radaelli, Marine Camus, Paraskevas Gkolfakis, Konstantinos Triantafyllou, Carlo Fabbri, Marta Patita, Erica Tan, Ellen Campbell, Michael Smyth, Hannah Beattie, Ravish Seeruthun, Alia Hadefi, Gabriela Rodríguez-Francisco, João Pedro Paulo, Luísa Gonçalves, Isabel Caetano, Alberto Savino, Marie Goudot, Antonia Panagaki, Eleni Koukoulioti, Maroulla Nikolaki, Giulia Gibiino, Alberto Gattuso, Ivo Mendes, Francisco Piçarra, Jorge Fonseca

**Affiliations:** a Gastroenterology Department, Hospital Garcia de Orta, Unidade Local de Saúde Almada-Seixal, Almada, Portugal; b Aging Lab, Egas Moniz Center for Interdisciplinary Research (CiiEM), Egas Moniz School of Health and Science, Almada, Portugal; c Gastroenterology Department, Ulster Hospital, Belfast, Northern Ireland, United Kindgom; d Department of Gastroenterology, Hepatopancreatology, and Digestive Oncology, Hopital Erasme, Hopital Universitaire de Bruxelles, ULB, Brussels, Belgium; e Gastroenterology and Hepatology, Hospital Universitario Ramón y Cajal, Madrid, Spain; f Instituto Ramón y Cajal de Investigación Sanitaria (IRYCIS), Madrid, Spain; g Centro de Investigación Biomédica en Red de Enfermedades Hepáticas y Digestivas (CIBERehd), Instituto de Salud Carlos III, Madrid, Spain; h Gastroenterology Department, Centro Hospitalar Universitário de Santo António, Unidade Local de Saúde de Santo António, Porto, Portugal; i Gastroenterology Department, Hospital de Santo André, Unidade Local de Saúde da Região de Leiria, Leiria, Portugal; j Gastroenterology Department, Valduce Hospital, Como, Italy; k Sorbonne University & CRSA (Centre de Recherche Saint Antoine), Endoscopy Unit, Saint Antoine Paris Hospital (APHP), Paris, France; l Department of Gastroenterology, Konstantopoulio-Patision General Hospital, Athens, Greece; m 2nd Department of Gastroenterology, Medical School, National and Kapodastrian University of Athens, Attikon University General Hospital, Athens, Greece; n Gastroenterology and Digestive Endoscopy Unit, Forlì-Cesena, AUSL Romagna, Italy; o Service de Gastroentérologie, CHU Nimes, France; p Department of Medical and Surgical Sciences, University of Bologna, Bologna, Italy

**Keywords:** Lower gastrointestinal bleeding, Ageing, Multimorbidity, Mortality

## Abstract

**Background:**

Population ageing in Europe is reshaping the clinical profile and outcomes of lower gastrointestinal bleeding (LGIB), but age-related comparative data remain scarce. We aimed to compare clinical presentation, management and 30-day outcomes between older and younger adults with LGIB.

**Methods:**

This retrospective, multinational, cohort study included consecutive adults presenting to emergency departments with LGIB between January 1 and December 31 in 2024. European hospitals routinely managing LGIB were eligible to participate. Ethical approval was obtained at hospital level. Patients were categorised in two age groups (≥65 and <65 years). The primary outcome was 30-day mortality.

**Findings:**

Overall, 1058 patients from 11 centres in seven European countries were included. Of these, 77.3% (818/1058) were aged ≥65 years and demonstrated a higher Oakland (21.0 ± 7.15), ABC (4.0 ± 2.9), and ALIBI (8.94 ± 3.7) scores, and a higher transfusion rate (50.9%, 416/818). Aetiology differed by age, with anorectal and inflammatory bowel diseases more common in younger adults and diverticular bleeding predominating in older patients. Endoscopy was performed in most patients (84.9%, 899/1058) and the rates of endoscopic therapy, interventional radiology, and surgery were similar across groups. Overall, 30-day mortality was 11.7% (124/1058) and was higher in older adults (13.7%, 112/818 versus 5.0%, 12/240), mainly due to non-bleeding-related causes (89.3%, 100/112). In multivariable analyses, ALIBI score (OR = 1.26 per-point, 95% CI 1.14–1.39), ABC score (OR = 1.25 per-point, 95% CI 1.15–1.36), and Charlson Comorbidity Index (OR = 1.25 per-point, 95% CI 1.14–1.37) were independently associated with 30-day mortality (p < 0.001). Age was inversely associated with intensive care unit admission (OR = 0.95 per-year, 95% CI 0.92–0.98; p = 0.0028).

**Interpretation:**

LGIB in older adults presents distinct clinical features with more severe bleeding. Higher baseline vulnerability might explain the age-related differences in escalation of care and worse outcomes. This supports the need for better integrated pathways of care in ageing European populations.

**Funding:**

FCT–10.13039/501100001871Fundação para a Ciência e a Tecnologia.

**Research in context**

**Evidence before this study**

Population ageing in Europe is reshaping the epidemiology of acute medical conditions, including lower gastrointestinal bleeding (LGIB). Older adults are known to have multiple comorbidities and higher use of antithrombotic therapy, which might have an impact on presentation and outcomes of LGIB in this age group. However, the extent to which these factors influence the contemporary clinical phenotype and outcomes of LGIB is not completely defined. Prior to this study, we conducted an extensive literature search in PubMed/MEDLINE, Embase, Web of Science, Scopus, and reference lists of relevant guidelines, reviews, and book chapters from inception up to December 31, 2025, without language restrictions. Search terms included combinations of “lower gastrointestinal bleeding”, “LGIB”, “haematochezia”, “melena”, “older adults”, “elderly”, “ageing”, “multimorbidity”, “frailty”, “antithrombotic therapy”, “mortality”, “readmission”, and “outcomes”. We considered population-based studies, multicentre cohorts, national audits, clinical guidelines, and studies evaluating risk scores or outcomes in acute LGIB. Studies focused exclusively on upper gastrointestinal bleeding, paediatric populations, occult bleeding, isolated iron deficiency anaemia without overt bleeding, or highly selected disease-specific cohorts were excluded. Existing evidence was heterogeneous in study design, LGIB definitions, age thresholds, management pathways, and outcome reporting, with most studies not primarily designed to compare older and younger adults. Risk of bias mainly reflected retrospective design, hospital-based selection, and incomplete adjustment for comorbidity, frailty, and antithrombotic exposure.

**Added value of this study**

This multicentre real-world cohort across seven European countries provides a comprehensive comparison between older and younger adults with LGIB. It shows that LGIB in older adults presents with distinct clinical features, characterised by different etiological patterns, higher bleeding risk scores, and substantially worse 30-day outcomes. Mortality in older patients was predominantly driven by non-bleeding-related causes, suggesting that LGIB often acts as a trigger for decompensation of other underlying diseases. This study identified conditions associated with higher mortality in this group which included greater comorbidities and higher bleeding risk scores. It also suggested endoscopic haemostatic therapy as a protective factor as it was associated with shorter length of stay. Age was inversely associated with intensive care unit admission, raising the possibility of age-related differences in escalation of care. Collectively, these findings support the conceptualisation of LGIB in older adults as a manifestation of systemic vulnerability rather than solely as an isolated gastrointestinal event. Indeed, some features may be compatible with a geriatric syndrome-like presentation, although no formal frailty or functional status assessment was performed.

**Implications of all the available evidence**

Taken together, the available evidence suggests that LGIB in older adults should be regarded not merely as an acute bleeding episode but also as a manifestation of underlying clinical vulnerability. Management strategies should therefore extend beyond haemostatic control to incorporate multimorbidity, frailty, medication review, and patient-centred goals of care, including careful consideration of escalation decisions. As European populations continue to age, age-adapted risk stratification, multidisciplinary care pathways, and prospective studies specifically targeting older populations are needed to inform guidelines, optimise resource allocation, and improve outcomes.

## Introduction

Lower gastrointestinal bleeding (LGIB) is a common medical emergency and an increasing clinical and organisational challenge for healthcare systems.^1^ Although traditionally considered less severe than upper gastrointestinal bleeding (UGIB), LGIB accounts for 20–30% of acute gastrointestinal haemorrhage presentations and is associated with substantial morbidity and healthcare resource use.^2^, ^3^, ^4^ Contemporary European population-based studies indicate that the case-mix of LGIB has undergone marked demographic and clinical shifts, largely driven by population ageing and the growing burden of chronic disease.^3^^,^^4^ Indeed, despite advances in pharmacological/endoscopic therapy, this condition is associated with longer hospitalisation and higher healthcare resource use when compared with UGIB.^5^

Ageing is closely associated with multimorbidity, frailty, and polypharmacy,^6^^,^^7^ all of which influence the presentation, severity, and outcomes of gastrointestinal bleeding. Older adults frequently have multiple chronic conditions that increase vulnerability to bleeding and challenge diagnostic and therapeutic decision-making.^8^ In parallel, the widespread use of antiplatelet and anticoagulant therapies has contributed substantially to the rising incidence of LGIB.^9^, ^10^, ^11^ In some contemporary European cohorts, more than half of patients presenting with LGIB are receiving at least one antithrombotic agent.^9^^,^^12^

Despite these trends, important knowledge gaps remain. In contrast to UGIB, age-specific data describing clinical phenotype, management patterns, and short-term outcomes in LGIB are limited, and most evidence derives from single-centre or single-country analyses with limited external validity.^3^ Moreover, few contemporary studies have examined the combined impact of ageing, multimorbidity, and antithrombotic exposure on LGIB outcomes. Indeed, current guidelines provide limited guidance tailored to this increasingly complex population,^13^^,^^14^ which is urgently needed.

To address these gaps, we aimed to compare clinical characteristics, bleeding aetiology, management, and short-term outcomes between older (≥65 years) and younger (<65 years) adults presenting with LGIB in contemporary European practice. We used age as a clinically relevant stratification variable to characterise differences in patient vulnerability, disease phenotype, and healthcare outcomes, and to identify predictors of adverse short-term outcomes.

## Methods

### Study design and setting

We conducted a retrospective, multinational, pan-European observational cohort study that consecutively included all patients admitted to the emergency department with LGIB. The full study protocol is available online. Participating centres were tertiary or secondary hospitals with gastroenterology departments and access to emergency endoscopy services, routinely managing patients with acute gastrointestinal bleeding. Centres were identified and invited through an existing European collaborative network of investigators with experience in gastrointestinal bleeding research. Selection was based on the ability to provide consecutive patient inclusion, reliable electronic medical records, and availability of 30-day follow-up data.

Given the observational design, there were no changes to patient care. Patient management was not standardised by the study protocol and followed routine clinical practice at each participating centre, informed by guideline recommendations.^13^ We followed the STROBE statement recommendations^15^ and the checklist is included in Supplementary Table S1.

### Participants

Adults aged ≥18 years with recent (<3 days) overt LGIB admitted to participating centres during a one full year enrolment period (January 1st–December 31st, 2024) were eligible. LGIB was defined as presumed to originate distal to the ileocaecal valve, presenting as haematochezia and/or melaena. All patients presenting with melaena underwent upper gastrointestinal endoscopy. Patients were excluded if, after review of clinical records, there was no evidence of overt LGIB. This group included patients investigated for isolated iron deficiency anaemia without overt bleeding or patients initially suspected of having LGIB but in whom clinical history and subsequent assessment did not support overt LGIB. We also excluded patients with confirmed UGIB on endoscopy.

### Data collection

Data was collected from the electronic medical records used in each hospital. This included the data related to the admission episode of the index LGIB and data related to further episodes that occurred in the 30 days after. Standardised data definitions and a common data dictionary were developed by the study coordinating committee and distributed to all participating centres within the study protocol. Unidentified data were entered in a secure electronic database with automated range checks and logic validation. Thirty-day outcomes were primarily assessed through electronic health record review. When follow-up information was unavailable within institutional records, additional follow-up was performed according to local practice and regulatory requirements through contact with patients, caregivers, or primary care physicians.

Collected variables included demographics, comorbidities (including cirrhosis, congestive heart failure, and diabetes), Charlson Comorbidity Index (CCI), previous LGIB, antiplatelet and anticoagulant therapy, clinical presentation (melaena, haematochezia, haematochezia + melaena), haemodynamic status, laboratory parameters at admission (haemoglobin, platelet count, international normalised ratio), LGIB aetiology, and endoscopic haemostatic therapy.

Bleeding risk scores (Oakland, age-blood tests-comorbidities [ABC], and acute lower gastrointestinal bleeding and in-hospital mortality [ALIBI]) were calculated as previously described.^16^^,^^17^

### Outcomes

The primary outcome was 30-day mortality following the index LGIB episode, counting from the day the patient presented to hospital.

Secondary outcomes included hospital and intensive care unit (ICU) admission, length of hospital stay, 30-day readmission, in-hospital mortality, need for red blood cell transfusion and requirement for endoscopic, radiological, or surgical intervention.

### Definitions

Patients were divided in two groups based on their age: older (≥65 years) and younger (<65 years). The age threshold of 65 years was prespecified and chosen in accordance with widely accepted definitions of older age in epidemiological, geriatric and clinical research.^18^ Red blood cell transfusion decisions were guided by haemoglobin level, haemodynamic status, ongoing bleeding, cardiovascular comorbidity, and overall clinical condition according to guideline recommendations.^13^ Haemodynamic instability was defined using the first available vital signs at emergency department presentation or hospital admission as systolic blood pressure <90 mmHg, heart rate >100 beats per minute, or requirement for vasopressor support. Rebleeding was defined as recurrent overt LGIB, new haemodynamic instability, or haemoglobin decrease ≥2 g/dL. Hospital admission followed local practice informed by guideline recommendations^13^ and was generally guided by bleeding severity, haemodynamic status, comorbidity burden, need for red blood cell transfusion or any endoscopic/radiology/surgical intervention, and discharge safety using the Oakland score. Readmission was defined as any all-cause unplanned hospital admission occurring within 30 days after discharge from the index LGIB hospitalisation, including but not limited to recurrent LGIB. Planned admissions, elective procedures, and scheduled follow-up admissions were not considered readmissions. Cause of death was classified from electronic medical records as bleeding-related or non-bleeding-related. Bleeding-related deaths were those directly attributed to uncontrolled or recurrent LGIB, haemorrhagic shock, or complications of bleeding management; all other deaths were classified as non-bleeding-related.

### Statistical analysis

The study sample size was defined by the inclusion of all consecutive eligible adult patients presenting with LGIB across participating centres during the predefined 12-month period. No formal sample size calculation was performed, as this was an observational real-world cohort study designed to capture the contemporary epidemiology and outcomes of LGIB. Accordingly, the final sample size reflects the total number of eligible patients identified during the study period.

Baseline characteristics were summarised using descriptive statistics. Continuous variables are presented as mean (SD) or median (IQR), as appropriate, and categorical variables as counts (percentages). Distributional assumptions for continuous variables were assessed using visual inspection of histograms and Q–Q plots, complemented by Shapiro–Wilk or Kolmogorov–Smirnov tests when appropriate. Between-group comparisons were performed using t tests or Mann–Whitney U tests for continuous variables and χ^2^ or Fisher's exact tests for categorical variables.

Missing predictor data were handled using multiple imputation by chained equations (MICE), generating 20 imputed datasets with 20 iterations each. Outcomes were not imputed but were included as auxiliary variables to improve the prediction of missing covariate values. Before imputation, the pattern of missingness was assessed according to country, age group, sex, clinical severity markers, and observed outcomes. As missing data were mainly concentrated in the ABC score and followed a country-dependent pattern, country was included in the imputation model as a non-imputed auxiliary categorical variable, thereby supporting the missing-at-random assumption conditional on the observed data-collection structure. Country was used only to inform imputation and was not included as a covariate in the final regression models. All models were fitted separately within each imputed dataset and pooled according to Rubin's rules.

Binary outcomes were analysed using logistic regression and reported as odds ratios (ORs) with 95% confidence intervals (CIs). Count outcomes with right-skewed distributions, including length of stay, were analysed using negative binomial regression and reported as incidence rate ratios (IRRs). Rare therapy strata were stabilised by collapsing low-frequency levels into an “Other” category. All statistical tests were two-sided.

For each outcome, a multivariable model was prespecified based on clinical relevance and prior literature and initially included age, sex, CCI, pharmacological therapies, endoscopic haemostatic intervention, and bleeding risk scores. Starting from this fully adjusted model, final parsimonious models were obtained by sequentially removing predictors with weak evidence of independent association, provided that their removal did not materially alter clinically relevant estimates. Model building avoided automated stepwise procedures based solely on statistical significance. Final model estimates were refitted within each imputed dataset and pooled.

Collinearity was assessed using correlation matrices and variance inflation factors. When substantial collinearity was identified, preference was given to retaining clinically interpretable variables over composite risk scores. Sensitivity analyses using ridge-penalised regression were performed to assess the robustness of estimates. To account for multiple testing, p values were adjusted using the Benjamini–Hochberg false discovery rate procedure.

Age was modelled as a continuous variable in multivariable analyses. In addition, restricted cubic splines were used as a sensitivity analysis to explore potential non-linear associations between age and outcomes. Spline models were compared with corresponding linear age models using the Akaike information criterion (AIC), and non-linearity was assessed by testing whether spline terms improved model fit beyond a linear age effect.

As an exploratory age-threshold sensitivity analysis, age was also modelled as a continuous predictor using flexible spline terms, with sex included as a covariate. Sex-standardised marginal estimates were derived for the age interval near the 65-year cutoff and compared with those for younger and older age intervals. Pairwise contrasts between intervals were evaluated using simulation-based uncertainty estimation, with Holm correction applied within each outcome. This analysis was performed to assess the stability of the selected 65-year threshold and was not used to redefine the main age groups.

To address the hierarchical structure of the cohort, an additional exploratory centre/country clustering sensitivity analysis was performed. Mixed effects generalised models were fitted with age group (≥65 versus <65 years) as the main fixed effect and sex as an adjustment covariate, including random intercepts for country and for participating centre nested within country. Binary endpoints were analysed using mixed-effects logistic models, whereas count endpoints were analysed using log-link mixed-effects count models.

### Ethics approval

The study was conducted in accordance with the Declaration of Helsinki and approved by the Ethics Committee of all centres involved (approval number 08/2023). All patient information was confidential in this study, and the requirement for informed consent was waived due to the study design and data anonymization.

### Role of the funding source

The study sponsor, FCT–Fundação para a Ciência e Tecnologia, had no role in the study design; collection, analysis, and interpretation of data; in the writing of the report; and the decision to submit the paper for publication.

## Results

**Supplementary Figure S1:**
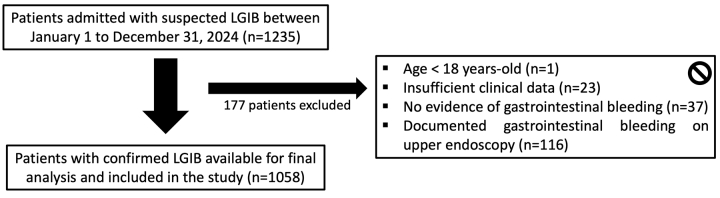
Patient flowchart. No evidence of GIB: absence of documented overt gastrointestinal bleeding after clinical record review, including isolated iron deficiency anaemia without haematochezia or melaena.

The patient flowchart of this study is detailed in Supplementary Figure S1.

### Baseline patient characteristics

Data from 1235 patients were collected across 11 participating centres in Europe: three from Portugal, two from Greece, two from Italy, one from Spain, Ireland, France and Belgium. Centre characteristics are included in Supplementary Table S2. From 1235 patients, 177 were excluded due to insufficient clinical data, no evidence of LGIB and/or documented UGIB cause on upper endoscopy. Data from 1058 patients were analysed. Overall, mean age was 73 ± 16 years (range 18–101), and 77.3% (818/1058) were aged ≥65 years; 53.7% (568/1058) were male.

Older adults had a higher comorbidity burden, particularly reflecting greater prevalence of congestive heart failure (OR 8.95) and chronic kidney disease (OR 4.15). Antiplatelet and anticoagulant therapy were also more common in this group, with aspirin and direct oral anticoagulants being the most frequently used agents. Regarding anticoagulation, 80.9% (17/21) younger and 89.5% (272/304) older adults had their therapy temporary withheld, in most cases (n = 13 and n = 168, respectively) due to the haemorrhagic risk and/or unclear indication. These therapies were resumed during hospitalisation in 82.4% (14/17) of younger patients and in 73.9% (201/272) older patients.

Previous LGIB episodes were reported in approximately one quarter of patients in both age groups. Baseline characteristics are summarised in Table 1.

**Table 1:** Baseline demographic and clinical characteristics according to age group.

Variables	<65 years (n = 240)	≥65 years (n = 818)	p value
Age (SD)	48 ± 13	80 ± 8	
Sex			
Female	93 (38.8%)	397 (48.5%)	0.003
Male	147 (61.2%)	421 (51.5%)	
Smoking: pack/year (SD)	8 ± 15.9	8 ± 20	0.156
Alcohol consumption: g/day (SD)	45.0 ± 38.3	44.9 ± 40.1	0.325
Comorbidities			
Charlson comorbidity index (SD)	1.75 ± 2.1	5.98 ± 2.9	<0.001
Liver cirrhosis	12 (5%)	50 (6.1%)	0.51
Congestive heart failure	12 (5%)	262 (32%)	<0.001
Ischaemic heart disease	22 (9.2%)	200 (24.4%)	<0.001
Diabetes mellitus	30 (12.5%)	229 (28%)	<0.001
Chronic kidney disease	16 (6.7%)	187 (22.9%)	<0.001
Medication at admission			
Antiplatelets	38 (15.8%)	304 (37.2%)	<0.001
Acetylsalicylic acid	22 (9.2%)	212 (25.9%)	<0.001
Clopidogrel	8 (3.3%)	46 (5.6%)	0.08
Dual antiplatelet therapy	8 (3.3%)	42 (5.1%)	0.124
Anticoagulants	21 (8.8%)	304 (37.2%)	<0.001
VKA	3 (1.3%)	45 (5.5%)	0.003
LMWH	4 (1.7%)	22 (2.7%)	0.184
DOAC	14 (5.8%)	238 (29.1%)	<0.001
Previous LGIB	51 (21.3%)	192 (23.5%)	0.236

SD, standard deviation; VKA, vitamin K antagonists; LMWH, low molecular weight heparin; DOAC, direct oral anticoagulants; LGIB, lower gastrointestinal bleeding.

### Clinical presentation and aetiology

Haematochezia was the most common presenting symptom in both groups. Melaena was less frequent overall but more common among older patients. Haemodynamic instability occurred in 10.8% of younger and 15.0% of older adults. Older patients presented lower haemoglobin levels at admission and higher bleeding risk scores–Oakland, ABC, and ALIBI (Table 2).

**Table 2:** Bleeding episode characteristics according to age group.

Variables	<65 years (n = 240)	≥65 years (n = 818)	p value
Clinical presentation			
Haematochezia	214 (89.2%)	652 (79.7%)	<0.001
Melaena	17 (7.1%)	117 (14.3%)	0.0021
Haematochezia + Melaena	9 (3.7%)	49 (6.0%)	0.107
Haemodynamic status			
Haemodynamic instability	26 (10.8%)	123 (15.0%)	0.049
Haematologic status			
Haemoglobin (SD), g/dL	11.4 ± 3.3	10.0 ± 3.77	<0.001
INR (SD)	1.12 ± 0.3	1.43 ± 1.53	0.005
Platelet count (SD), ×10^9^/L	276.0 ± 115.4	242 ± 110.5	<0.001
Risk scores			
Oakland score	15.7 ± 7.6	21.0 ± 7.15	<0.001
ABC score	1.44 ± 2.4	4.0 ± 2.9	<0.001
ALIBI score	3.6 ± 2.7	8.94 ± 3.7	<0.001
LGIB cause			
Angiodysplasia	3 (1.3%)	72 (8.8%)	<0.001
Anorectal diseases	61 (25.4%)	102 (12.5%)	<0.001
Diverticulosis	13 (5.4%)	195 (23.8%)	<0.001
Inflammatory bowel disease	36 (15.0%)	10 (1.2%)	<0.001
Infectious colitis	9 (3.8%)	14 (1.7%)	0.0287
Undetermined colitis	10 (4.2%)	21 (2.6%)	0.0985
Ischaemic proctitis/colitis	18 (7.5%)	94 (11.5%)	0.0383
Radiation proctitis/colitis	1 (0.4%)	26 (3.2%)	0.00842
Colorectal polyps	6 (2.5%)	10 (1.2%)	0.0764
Postpolypectomy bleeding	17 (7.1%)	41 (5.0%)	0.107
Colorectal cancer	15 (6.3%)	70 (8.6%)	0.123
Other	22 (9.2%)	41 (5.0%)	0.00842
Inconclusive	28 (11.7%)	122 (14.9%)	0.102

SD, standard deviation; INR, international normalized ratio; LGIB, lower gastrointestinal bleeding; Other, rare non-prespecified causes, including malignancy-related colonic involvement, neutropenic enterocolitis and post-surgical bleeding after colorectal surgery.

**Fig. 1:**
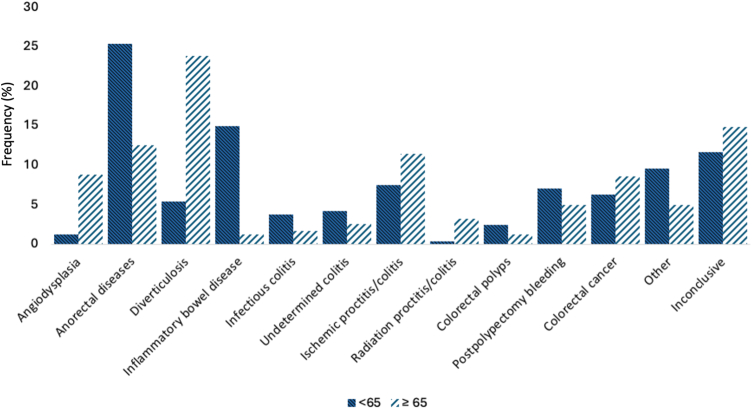
Aetiology of lower gastrointestinal bleeding according to age group. Frequencies are presented as percentages within each age group.

LGIB causes differed by age. Anorectal disorders (25.4%) and inflammatory bowel disease (15.0%) predominated in younger adults, whereas diverticulosis (23.8%) was the leading cause among older patients. Angiodysplasia (8.8%), ischaemic colitis (11.5%), and radiation proctitis (3.2%) were largely confined to older individuals (Fig. 1).

### Diagnostic workup and inpatient interventions

Red blood cell transfusion was more frequent among older adults (approximately 50%). Lower endoscopic evaluation was performed in most patients (90% of younger and 83.4% of older adults), with a median time to procedure of 1 day in both groups (IQR 1–6.25 versus IQR 1–3, respectively). Therapeutic endoscopy was performed in 18.3% of younger and 19.9% of older patients. Through-the-scope (TTS) clip was the most common endoscopic haemostatic method in the former group (38.6%, 17/44), followed by combination therapy (22.7%, 10/44), which contrasts with the older group, in which argon plasma coagulation (39.1%, 63/161) was the preferred method, followed by TTS clip (25.4%, 41/161). These haemostatic modalities differed according to bleeding aetiology: APC was predominantly used for angiodysplasia and radiation proctitis/colitis, whereas TTS clips were mainly used for post-polypectomy bleeding and focal anorectal or diverticular bleeding (p < 0.001).

Overall, 9.6% (23/240) younger adults had no lower gastrointestinal endoscopy, in two cases considered to be palliative care only (and died during hospitalisation); 16.4% (134/818) older patients also did not undergo endoscopic evaluation, of which 39 died while hospitalised. Interventional radiology and surgery were infrequent and did not differ between age groups (Table 3).

**Table 3:** Inpatient interventions and outcomes for lower gastrointestinal bleeding according to age group.

Variables	<65 years (n = 240)	≥65 years (n = 818)	p value
Inpatient interventions			
Red blood cell transfusion (any)	60 (25.0%)	416 (50.9%)	<0.001
Received 1–2 units	26 (10.8%)	248 (30.3%)	<0.001
Received 3–4 units	17 (7.1%)	114 (13.9%)	<0.001
Received >4 units	17 (7.1%)	54 (6.6%)	0.397
Fresh frozen plasma	4 (1.7%)	11 (1.3%)	0.356
Prothrombin complex concentrate	1 (0.4%)	6 (0.7%)	0.298
Endoscopy performed	216 (90.0%)	683 (83.4%)	0.007
Rectosigmoidoscopy	88 (36.7%)	223 (27.2%)	0.003
Colonoscopy	128 (53.3%)	460 (56.2%)	0.212
Haemostatic endoscopic therapy	44 (18.3%)	161 (19.9%)	0.322
Computed tomography angiography	57 (23.8%)	188 (23%)	0.401
Embolisation	3 (1.3%)	4 (0.5%)	0.100
Surgery in the first 30 days	7 (2.9%)	24 (2.9%)	0.996
Outcomes			
Hospital admission	149 (62.1%)	689 (84.2%)	<0.001
Length of stay (days)	9.1 ± 11.5	12.2 ± 17.9	0.016
ICU admission	22 (9.2%)	38 (4.6%)	0.004
Rebleeding	22 (9.2%)	115 (14.1%)	0.024
Readmission	15 (6.3%)	72 (8.8%)	0.022
In-hospital mortality	12 (5%)	83 (10.1%)	0.007
30-day mortality	12 (5%)	112 (13.7%)	<0.001

### Patient outcomes

**Supplementary Figure S2:**
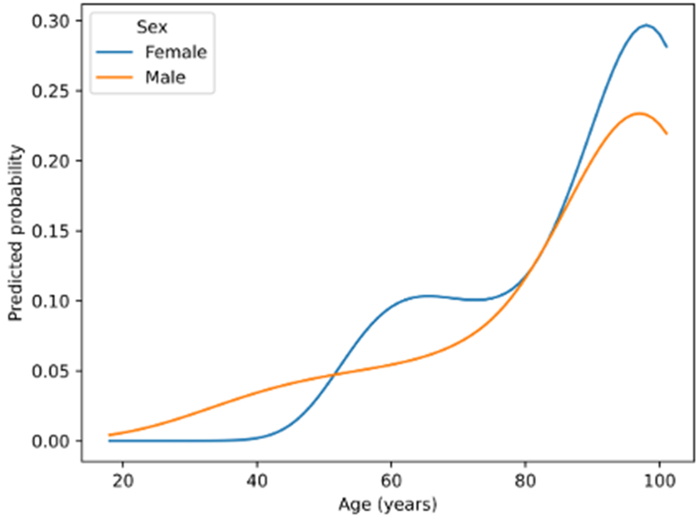
Predicted probability of 30-day mortality according to age, modelled using restricted cubic splines and stratified by sex.

Overall, in-hospital mortality was 9.0% (95/1058) and 30-day mortality was 11.7% (124/1058). Mortality was more than twice as high among older adults and was predominantly attributable to non-bleeding-related causes (89.3%, 100/112). These deaths were most frequently attributed to cardiovascular events (17.9%, 20/112), sepsis with multiorgan failure (15.2%, 17/112) and respiratory infections (13.4%, 15/112). LGIB-related deaths accounted for 10.7% (12/112). Specific causes of death according to age group are depicted in Supplementary Table S3. The association between age and 30-day mortality stratified by sex is shown in Supplementary Figure S2.

Older patients had higher rates of hospital admission, longer length of stay, and greater 30-day readmission. In contrast, ICU admission was more frequent among younger adults. Rebleeding occurred in 22 younger and 115 older patients, mostly associated with anorectal disease (54.5%, 12/22) and diverticular bleeding (53.9%, 62/115), respectively.

On multivariate analysis (Table 4), across outcomes, bleeding risk scores were the dominant independent predictors. For disposition endpoints, both Oakland and ABC scores were independently associated with hospital (Oakland: OR = 1.10 per-point; ABC: OR = 1.18 per-point) and ICU admission (Oakland: OR = 1.12 per-point; ABC: OR = 1.45 per-point). Age was inversely associated with ICU admission (OR 0.95 per-year).

**Table 4:** Predictors of binary outcomes on multivariable logistic regression and count outcomes (length of stay and total blood transfusions) on log-link count models.

Outcome	N with observed outcome (events)	Predictor	Adjusted OR/IRR (95% CI)	p value (FDR)
Hospital admission	1058 (838)	Oakland score (per 1-point increase)	1.10 (1.07–1.13)	<0.001
		ABC score (per 1-point increase)	1.18 (1.07–1.30)	0.0035
ICU admission	1058 (60)	ABC score (per 1-point increase)	1.45 (1.24–1.69)	<0.001
		Oakland score (per 1-point increase)	1.12 (1.06–1.18)	<0.001
		Age (per 1-year increase)	0.95 (0.92–0.98)	0.0028
Readmission	1056 (87)	None (no predictors retained significance after FDR correction)	–	–
In-hospital mortality	1057 (95)	ALIBI score (per 1-point increase)	1.32 (1.21–1.44)	<0.001
		ABC score (per 1-point increase)	1.30 (1.18–1.42)	<0.001
		Charlson Comorbidity Index (per 1-point increase)	1.19 (1.07–1.32)	0.0068
30-day mortality	1056 (123)	ALIBI score (per 1-point increase)	1.26 (1.14–1.39)	<0.001
		ABC score (per 1-point increase)	1.25 (1.15–1.36)	<0.001
		Charlson Comorbidity Index (per 1-point increase)	1.25 (1.14–1.37)	<0.001
Surgery in the first 30 days	1056 (31)	ALIBI score (per 1-point increase)	0.79 (0.69–0.91)	0.0096
Length of stay	1052	ALIBI score (per 1-point increase)	1.05 (1.04–1.06)	<0.001
		ABC score (per 1-point increase)	1.09 (1.05–1.13)	<0.001
		Antiplatelet therapy: Clopidogrel (versus Aspirin)	1.60 (1.10–2.32)	0.0458
		Haemostatic endoscopic therapy: Yes (versus No)	0.78 (0.64–0.95)	0.0458
Total blood transfusions	1056	ALIBI score (per 1-point increase)	1.13 (1.12–1.15)	<0.001
		ABC score (per 1-point increase)	1.09 (1.05–1.13)	<0.001
		Anticoagulant therapy: Other (versus DOAC)	1.83 (1.33–2.50)	<0.001

Odds ratios (OR) are expressed per 1-unit increase for continuous predictors. Incidence rate ratios (IRR) are expressed per 1-unit increase for continuous predictors. FDR, false discovery rate.

For mortality, ALIBI score was the strongest independent predictor of both in-hospital (OR 1.32 per-point) and 30-day mortality (OR 1.26 per-point). CCI was also independently associated with in-hospital (OR = 1.19 per-point) and 30-day mortality (OR = 1.25 per-point).

Length of stay increased with ALIBI (IRR = 1.05 per-point) and ABC (IRR = 1.09 per-point) scores and with clopidogrel use (IRR = 1.60), whereas therapeutic endoscopic intervention was associated with shorter hospitalisation (IRR = 0.78). Transfusion requirements were strongly associated with ALIBI (IRR = 1.13 per-point) and ABC (IRR = 1.09) scores and were higher among patients receiving vitamin K antagonists or low molecular weight heparin (IRR = 1.93) compared with direct oral anticoagulants.

Sex was included in the multivariable models but was not retained as an independent predictor of the main clinical outcomes after adjustment for comorbidity burden and bleeding risk scores.

As a sensitivity analysis, age was additionally modelled using restricted cubic splines (Supplementary Table S4). This approach did not materially improve model fit for most outcomes, including hospital admission, readmission, in-hospital and 30-day mortality, surgery, or transfusion outcomes, although some evidence of non-linearity was observed for ICU admission and length of hospital stay. Therefore, age was retained as a linear term in the primary multivariable models.

Missing predictor data were limited, with the highest proportion observed for ABC score (175/1058, 16.5%). Missingness was lower for Oakland score (33/1058, 3.1%), ALIBI score (29/1058, 2.7%), and haemostatic endoscopic therapy (9/1058, 0.9%), while age, sex, and CCI had no missing data. Missingness for binary outcomes was below 1.2% (Supplementary Table S5). The missingness pattern was not consistent with a completely random mechanism. Missing ABC score values were strongly country dependent. Patients with and without missing ABC score values had similar age, sex distribution, Oakland score, and ALIBI score, suggesting that missingness was mainly related to country-level data availability. This pattern was considered compatible with a missing-at-random assumption conditional on country and other observed variables.

In the exploratory age-threshold sensitivity analysis, the subgroup aged 60–64 years showed a clinical profile generally closer to the younger reference interval for 30-day mortality, ICU admission, readmission and red blood cell transfusion (Supplementary Table S6).

In an exploratory centre/country clustering sensitivity analysis, the main findings were directionally consistent with the primary analyses. Patients aged≥ 65 years had higher hospital admission (OR = 2.54, 95% CI 1.77–3.64), red blood cell transfusion (OR = 2.23, CI 1.60–3.11), longer length of stay (IRR = 1.45, CI 1.37–1.53), higher in-hospital mortality (OR = 1.91, CI 1.04–3.50) and 30-day mortality (OR = 2.83, CI 1.54–5.22). ICU admission remained less frequent in older patients (OR = 0.54, CI 0.30–0.96). These results are reported in Supplementary Table S7.

## Discussion

In this large pan-European cohort, LGIB in older adults emerged with distinct clinical features, characterised by greater comorbidities, higher exposure to antithrombotic therapy, different etiological patterns, higher bleeding severity scores and markedly worse short-term outcomes. Importantly, mortality was predominantly attributed to non-bleeding-related causes, showing that in older patients the bleeding episode frequently acts as a trigger for decompensation of chronic diseases rather than as the main prognostic determinant. In-hospital and 30-day fatality rates exceeded that reported in many previous cohorts.^19^ Compared with in-hospital mortality, 30-day mortality captures additional deaths occurring after discharge and may therefore better reflect short-term vulnerability in older patients with multimorbidity and competing causes of deterioration. The observed distinct clinical features suggest an age-related phenotype that is best interpreted not as an effect of chronological age alone, but as a proxy for accumulated vulnerability characterised by multimorbidity, polypharmacy, and frailty that becomes increasingly prevalent with ageing.^20^ Furthermore, the high proportion of older adults likely reflects both the epidemiology of LGIB in ageing European populations and possible selection factors related to inclusion of tertiary and secondary hospitals with established gastrointestinal departments. This reinforces the relevance of studying LGIB as a condition increasingly occurring in older, multimorbid, and clinically vulnerable patients, while also requiring caution when generalising these proportions to all European healthcare settings.

The high prevalence of antithrombotic therapy reflects the higher cardiovascular burden of this population. While antiplatelet and anticoagulant agents are essential for cardiovascular prevention, they act as risk factors for LGIB in ageing populations.^21^ In our cohort, clopidogrel and vitamin K antagonists were independently associated with prolonged hospitalisation, underscoring how medication-related bleeding risk intersects with frailty and comorbidity to shape a not previously appreciated global clinical profile of LGIB. However, despite being substantially more frequent among older adults, anticoagulant therapy was not independently associated with in-hospital or 30-day mortality on multivariate analysis. This suggests that mortality in older patients may be more strongly driven by baseline vulnerability and systemic disease burden than by anticoagulant exposure alone. Antithrombotic therapy may contribute to the clinical context of LGIB, but adverse outcomes in this cohort seem to be more strongly related to comorbidity decompensation and bleeding risk scores than to antithrombotic therapy alone.

Older patients more frequently presented with haemodynamic instability and lower haemoglobin levels, likely reflecting more severe bleeding, delayed presentation, and reduced physiological reserve. Composite bleeding risk scores integrating baseline health status and laboratory parameters, particularly ALIBI, were strong independent predictors of mortality and length of stay. This reinforces the notion that risk stratification in modern LGIB must move beyond haemodynamic variables alone to capture systemic vulnerability. Scores that incorporate comorbidity and baseline clinical characteristics may therefore be particularly relevant in ageing populations. While the Oakland score was primarily developed to identify low-risk patients suitable for discharge,^22^ it shows limited calibration for adverse outcomes beyond this purpose. The ABC score, although initially derived for UGIB, has demonstrated reasonable performance in LGIB but remains relatively complex and lacks external validation.^13^ The ALIBI score, recently proposed, integrates bleeding severity and patient condition, aligning well with the needs of an ageing population.^23^ Although these scores were derived for specific clinical endpoints, their consistent associations with mortality and healthcare use in our cohort suggest that their prognostic utility may extend beyond their original validation context. Nevertheless, this observation should be interpreted cautiously and warrants prospective evaluation.

Age-related differences in aetiology further reflect this shift in modern LGIB. Diverticulosis, angiodysplasia, ischaemic colitis, and radiation proctitis/colitis predominated in older adults, reflecting age-related vascular fragility and the cumulative burden of chronic disease.^9^ In contrast, younger patients more commonly presented with anorectal disease and inflammatory bowel disease, entities less tightly linked to systemic decline. These different patterns suggest that LGIB increasingly represents a heterogeneous biological process across the age spectrum. In our cohort, different aetiologies further explain variations in haemostatic procedures and should therefore be interpreted as primarily lesion-dependent rather than directly age-dependent. Inconclusive aetiology is also a recognised challenge in LGIB,^1^^,^^19^ which reached almost 15% in older adults, and is often related to suboptimal bowel preparation, impaired visualisation from blood or clots, intermittent bleeding from diverticulosis or angiodysplasia with spontaneous cessation before colonoscopy, and occasionally undetected mid-gastrointestinal bleeding.

A particularly interesting finding was the inverse association between age and ICU admission, despite substantially higher mortality among older adults. Although causality cannot be inferred and unmeasured factors may have influenced decisions on ICU admission, this raises important questions regarding escalation of care in ageing populations. Decisions surrounding ICU admission should be guided by clinical severity rather than patients’ chronological age alone.^24^ As populations age, equitable access to escalation of care warrants careful consideration.

The observation that haemostatic endoscopic therapy was associated with shorter hospital stay is also clinically relevant. While intervention is typically reserved for significant bleeding, effective endoscopic control may mitigate downstream complications and facilitate recovery. This may suggest a higher than previously appreciated role in therapeutic endoscopy in LGIB.^13^ Notably, rates of therapeutic endoscopy, interventional radiology, and surgery were similar across age groups, despite worse outcomes among older adults. Our findings suggest endoscopic control alone is unlikely to address the full risk profile of older adults with LGIB. On the other hand, a higher proportion of normal findings on endoscopy seen in the older group reinforces the importance of careful selection of patients. Since advanced age is a risk factor for sedation and endoscopic-related adverse events, it is essential to balance the benefits versus risks before planning endoscopic evaluation.^25^ The association between haemostatic endoscopic therapy and length of stay should also be interpreted cautiously since it may reflect the presence of an identifiable and treatable bleeding source rather than a direct causal effect. Indeed, whether earlier diagnostic or haemostatic intervention can prevent systemic decompensation in clinically vulnerable older adults with LGIB remains uncertain and should be evaluated in prospective studies.

Collectively, these findings support a reconceptualisation of LGIB in older adults. Rather than an isolated gastrointestinal event, LGIB may represent a sentinel event within a trajectory of multimorbidity and frailty.^26^ Its interaction with physiological reserve, polypharmacy and systemic vulnerability may be consistent with a geriatric syndrome-like presentation, recently proposed for UGIB,^27^ although no formal frailty or functional status assessment was performed. This perspective has practical implications: management strategies should integrate comorbidity optimisation, medication review, haemostatic management when active or recent bleeding is identified and post-discharge follow-up. Indeed, our findings suggest that, in older adults, haemostatic control should be complemented by broader patient assessment given that adverse outcomes were often not directly attributed to LGIB, highlighting the complexity of these patients.^28^, ^29^, ^30^

The strengths of this study include its large real-world pan-European cohort, representation of diverse healthcare systems, and inclusion of data over a full calendar year enhancing generalisability and allowing capture of seasonal variations. Limitations include its retrospective design, restriction to 30-day outcomes and reliance on electronic medical records. Race and ethnicity were not available in a sufficiently complete or harmonised manner across participating centres and therefore could not be reliably analysed. Although 30-day outcomes were assessed using predefined definitions across centres, follow-up relied partly on electronic health records and local follow-up procedures, which may have introduced heterogeneity in outcome ascertainment. The inverse association between age and ICU admission may have been influenced by unmeasured factors, including frailty, functional status, treatment-limitation decisions, ICU availability and centre-specific policies. The timing of antithrombotic therapy resumption was not systematically captured, limiting assessment of its influence on rebleeding and mortality. Moreover, retrospective cause of death classification may be subject to misclassification, particularly in patients with multiple competing causes of deterioration. Systematic small-bowel evaluation was not performed in all patients with inconclusive findings, and it is plausible that few cases represented small bowel bleeding rather than genuine LGIB. The real-world design increases external validity but also introduces potential centre-level and country level heterogeneity and clustering, particularly for management-related outcomes. Moreover, participating centres were tertiary or secondary hospitals with established LGIB services which may introduce referral bias and may have contributed to the high rate of endoscopic evaluation found in our cohort; our findings may not be fully generalisable to smaller hospitals or community settings without gastroenterology departments. Frailty was not formally assessed, limiting our ability to evaluate its potential contribution to adverse outcomes in older patients. As an observational cohort study, the possibility of residual confounding remains, and causal relationships between age, management decisions, and outcomes cannot be inferred. Finally, although the ≥65-year threshold facilitates comparison with previous studies, chronological age remains an imperfect surrogate for biological ageing.

This study identifies several priorities for future research. First, prospective studies specifically designed for older populations are needed to better characterise the interaction between LGIB, multimorbidity, frailty, and polypharmacy including antithrombotic therapy and to determine whether LGIB should be formally conceptualised within the framework of geriatric syndromes. Second, future research should evaluate age-adapted management strategies including assessment of healthcare-system variation, risk stratification pathways and multidisciplinary care models, to optimise decision-making regarding hospital admission, endoscopic evaluation, and escalation of care. Alternative haemodynamic measures, including shock index, may provide additional prognostic information in future prospective studies. Third, further validation of contemporary bleeding risk scores in older populations is warranted, particularly to assess their performance across different healthcare systems and levels of clinical vulnerability. Finally, longitudinal studies assessing functional decline, readmission, and longer-term outcomes after LGIB would help clarify the broader clinical impact of this condition in ageing populations.

In conclusion, LGIB in ageing populations represents a shifting clinical paradigm. The observed distinct clinical features may result from the clinical expression of systemic vulnerability with multimorbidity that becomes increasingly prevalent with ageing. As Europe's population continues to age, LGIB in older adults should no longer be managed solely as an isolated bleeding event. Instead, it should prompt a broader clinical assessment incorporating multimorbidity, frailty, and medication exposure. Integrating these principles into LGIB care may represent an important step toward improving outcomes in ageing healthcare systems.

## Contributors

FVL: article conception, design, literature review and draft of the manuscript. CP, TT, MA, ERS, IP, DS, FR, MC, PG, KT, CF, MP, ET, EC, MS, HB, RS, AH, GRF, JPP, LG, IC, AS, MG, AP, EK, MN, GG, AG, IM, FP: patient recruitment and critical review of the article for important intellectual content. PM: statistical analysis and critical review of the article for important intellectual content. JF: article conception, design, critical review of the article for important intellectual content.

FVL, PM, JF had access to all the data. FVL and PM verified the underlying data. PM wrote all data management and analysis code. JF provided supervision.

FVL and JF were responsible for the decision to submit the manuscript.

## Data sharing statement

Data sharing request will be considered upon written request via email to the corresponding author, subject to approval by the relevant institutional and ethical authorities and in accordance with applicable data protection regulations.

## Declaration of interests

ERS reports grants from Instituto de Salud Carlos III, Asociación Española de Gastroenterología, Sociedad Española de Endoscopia Digestiva and 3D Matrix; honoraria from Olympus, Erbe, Fujifilm, Izasa and 3D Matrix; and payment for expert testimony from Olympus, Boston Scientific, Astrazeneca, Creo Medical and Adacyte Therapeutics outside of the presented work. FVL, CP, PM, TT, MA, IP, DS, FR, MC, PG, KT, CF, MP, ET, EC, MS, HB, RS, AH, GRF, JPP, LG, IC, AS, MG, AP, EK, MN, GG, AG, IM, FP and JF have no interests to declare.

## Appendix A Supplementary data

Supplementary Table S7

Study Protocol
