# Supplementary material for: Age-related differences in the presentation, management, and outcomes of lower gastrointestinal bleeding: a retrospective multinational cohort study
Source: Lancet Reg Health Eur. 2026 Jul 9;68:101775. doi: 10.1016/j.lanepe.2026.101775 (PMC13380016; doi:10.1016/j.lanepe.2026.101775)
Supplement: Supplementary Table S1 [file mmc1.pdf]

The STROBE reporting checklist

For checking that observational epidemiology research articles can be understood and used by everyone

	Item Description	Location (or reason for not reporting)
**Title and abstract**		
1a. Indicate the study's design	Indicate the study's design with a commonly used term in the title or the abstract.	Pages 1-2
1b. Abstract	Provide in the abstract an informative and balanced summary of what was done and what was found.	Page 2
**Introduction**		
2. Background / rationale	Explain the scientific background and rationale for the investigation being reported.	Page 4
3. Objectives	State specific objectives, including any prespecified hypotheses.	Page 4
**Methods**		
4. Study design	Present key elements of study design early in the paper.	Page 5
5. Setting	Describe the setting, locations, and relevant dates, including periods of recruitment, exposure, follow-up, and data collection.	Page 5
6a. Eligibility criteria	**Cohort study:** Give the eligibility criteria, and the sources and methods of selection of participants. Describe methods of follow-up. **Case-control study:** Give the eligibility criteria, and the sources and methods of case ascertainment and control selection. Give the rationale for the choice of cases and controls. **Cross-sectional study:** Give the eligibility criteria, and the sources and methods of selection of participants.	Page 5
6b. Matching criteria	**Cohort study:** For matched studies, give matching criteria and number of exposed and unexposed. **Case-control study:** For matched studies, give matching criteria and the number of controls per case.	Not applicable/performed
7. Variables	Clearly define all outcomes, exposures, predictors, potential confounders, and effect modifiers. Give diagnostic criteria, if applicable.	Pages 5-8

8. Data sources / measurement	For each variable of interest give sources of data and details of methods of assessment (measurement). Describe comparability of assessment methods if there is more than one group.	Pages 5-8
9. Bias	Describe any efforts to address potential sources of bias.	Pages 5-8
10. Study size	Explain how the study size was arrived at.	The study sample size was determined by including all consecutive adult patients presenting with lower gastrointestinal bleeding across participating centers during a predefined 12-month period. No formal sample size calculation was performed, as this was an observational real-world cohort study designed to capture the contemporary epidemiology and outcomes of LGIB. The final sample size reflects the total number of eligible patients during the study period.
11. Quantitative variables	Explain how quantitative variables were handled in the analyses. If applicable, describe which groupings were chosen, and why.	Pages 6-8
12a. Statistical methods	Describe all statistical methods, including those used to control for confounding.	Pages 7-8
12b. Statistical methods – subgroups and interactions	Describe any methods used to examine subgroups and interactions.	Pages 7-8
12c. Statistical methods – missing data	Explain how missing data were addressed.	Pages 7-8
12di. Statistical methods – loss to follow-up	**Cohort study:** If applicable, describe how loss to follow-up was addressed.	Pages 7-8
12dii. Statistical methods – matching cases and controls	**Case-control study:** If applicable, explain how matching of cases and controls was addressed.	Not applicable
12diii. Statistical methods – sampling strategy	**Cross-sectional study:** If applicable, describe analytical methods taking account of sampling strategy.	Not applicable

12e. Statistical methods – sensitivity analyses	Describe any sensitivity analyses.	Page 7-8
**Results**		
13a. Participant numbers	Report the numbers of individuals at each stage of the study—e.g., numbers potentially eligible, examined for eligibility, confirmed eligible, included in the study, completing follow-up, and analysed; Consider use of a flow diagram.	Page 8-9
13b. Participants – non-participation	Give reasons for non-participation at each stage.	Page 8-9
13c. Participants – flow diagram	Consider use of a flow diagram.	Figure 1 (supplementary material)
14a. Descriptive data – participant characteristics	Give characteristics of study participants (e.g., demographic, clinical, social) and information on exposures and potential confounders. Present the information in a table.	Page 7-8
14b. Descriptive data – missing data	Indicate the number of participants with missing data for each variable of interest.	Supplementary Table 4; Methods – Statistical analysis. Missing predictor data were handled using multiple imputation; outcomes were not imputed.
14c. Descriptive data – follow-up time	**Cohort study:** Summarise follow-up time—e.g., average and total amount.	Tables 1-4 (30-day follow-up)
15. Outcome data	**Cohort study:** Report numbers of outcome events or summary measures over time. **Case-control study:** Report numbers in each exposure category, or summary measures of exposure. **Cross-sectional study:** Report numbers of outcome events or summary measures.	Table 4
16a. Main results	Give unadjusted estimates and, if applicable, confounder-adjusted estimates and their precision (e.g., 95% confidence intervals). Make clear which confounders were adjusted for and why they were included.	Pages 8-11
16b. Main results – category boundaries	Report category boundaries when continuous variables were categorised.	Table 4 ; pages 8-11
16c. Main results – risk	If relevant, consider translating estimates of relative risk into absolute risk for a meaningful time period.	Table 4
17. Other analyses	Report other analyses done—e.g., analyses of subgroups and interactions, and sensitivity analyses.	Table 4
**Discussion**		

18. Key results	Summarise key results with reference to study objectives.	Pages 11-13
19. Limitations	Discuss limitations of the study, taking into account sources of potential bias or imprecision. Discuss both direction and magnitude of any potential bias.	Page 14
20. Interpretation	Give a cautious overall interpretation considering objectives, limitations, multiplicity of analyses, results from similar studies, and other relevant evidence.	Page 14-15
21. Generalisability	Discuss the generalisability (external validity) of the study results.	Page 15
**Other information**		
22. Funding	Give the source of funding and the role of the funders for the present study and, if applicable, for the original study on which the present article is based.	Page 15
