# Supplementary material for: Age-related differences in the presentation, management, and outcomes of lower gastrointestinal bleeding: a retrospective multinational cohort study
Source: Lancet Reg Health Eur. 2026 Jul 9;68:101775. doi: 10.1016/j.lanepe.2026.101775 (PMC13380016; doi:10.1016/j.lanepe.2026.101775)
Supplement: Supplementary Table S2 [file mmc2.docx]

| **Country** | **Hospital** | **Centre type** | **Endoscopy** | **Interventional radiology** | **General surgery** | **Gastroenterology availability** |
| --- | --- | --- | --- | --- | --- | --- |
| Belgium | Hôpital Erasme | Tertiary | Yes | Yes | Yes | 08:00-08:00 |
| France | Saint Antoine Paris Hospital | Tertiary | Yes | Yes | Yes | 08:00-08:00 |
| Greece | Attikon University General Hospital | Tertiary | Yes | Yes | Yes | 08:00-08:00 |
|  | Konstantopoulio-Patision General Hospital | Secondary | Yes | Yes | Yes | 08:00-08:00 |
| Italy | AUSL Romagna | Tertiary | Yes | Yes | Yes | 08:00-08:00 |
|  | Valduce Hospital | Secondary | Yes | Yes | Yes | 08:00-08:00 |
| Northern Ireland | Ulster Hospital | Tertiary | Yes | Yes | Yes | 08:00-17:00 |
| Portugal | Centro Hospitalar Universitário de Santo António | Tertiary | Yes | Yes | Yes | 08:00-08:00 |
|  | Hospital de Santo André | Secondary | Yes | Yes | Yes | 08:00-08:00 |
|  | Hospital Garcia de Orta | Tertiary | Yes | Yes | Yes | 08:00-20:00 |
| Spain | Hospital Universitario Ramón y Cajal | Tertiary | Yes | Yes | Yes | 08:00-08:00 |

**Supplementary table 2**: Gastroenterology department characterisation.
