# Supplementary material for: Age-related differences in the presentation, management, and outcomes of lower gastrointestinal bleeding: a retrospective multinational cohort study
Source: Lancet Reg Health Eur. 2026 Jul 9;68:101775. doi: 10.1016/j.lanepe.2026.101775 (PMC13380016; doi:10.1016/j.lanepe.2026.101775)
Supplement: Supplementary Table S3 [file mmc3.docx]

| **Variables** | **<65 years (n=240)** | **≥65 years (n=818)** |
| --- | --- | --- |
| 30-day mortality | 12 (5%) | 112 (13.7%) |
| Primary cause of death | | |
| Due to LGIB | 2 (16.7%) | 12 (10.7%) |
| Gastrointestinal malignancy | 0 (0.0%) | 7 (6.3%) |
| Non-gastrointestinal malignancy | 3 (25.0%) | 14 (12.5%) |
| Sepsis with multiorgan failure | 4 (33.3%) | 17 (15.2%) |
| Pneumonia | 0 (0.0%) | 15 (13.4%) |
| Myocardial infarction, pulmonary thromboembolism, decompensated heart failure, cardiorespiratory arrest | 0 (0.0%) | 20 (17.9%) |
| Stroke | 0 (0.0%) | 6 (5.4%) |
| Decompensated liver disease | 2 (16.7%) | 2 (1.8%) |
| Chronic kidney disease | 0 (0.0%) | 5 (4.5%) |
| Urinary tract infection | 1 (8.3%) | 4 (3.6%) |
| Other | 0 (0.0%) | 5 (4.5%) |
| Unknown | 0 (0.0%) | 5 (4.5%) |

**Supplementary table 3**: causes of death of patients with lower gastrointestinal bleeding. LGIB – lower gastrointestinal bleeding.

Other: meningitis, dementia, infected pressure sore.
