# Supplementary material for: Age-related differences in the presentation, management, and outcomes of lower gastrointestinal bleeding: a retrospective multinational cohort study
Source: Lancet Reg Health Eur. 2026 Jul 9;68:101775. doi: 10.1016/j.lanepe.2026.101775 (PMC13380016; doi:10.1016/j.lanepe.2026.101775)
Supplement: Supplementary Table S4 [file mmc4.docx]

| **Outcome** | **N/events** | **ΔAIC spline vs. linear** | **Non-linearity p value** | **Interpretation** |
| --- | --- | --- | --- | --- |
| Hospital admission | 1058 / 838 | +3.14 | 0.652 | No improvement |
| ICU admission | 1045 / 60 | −12.07 | 0.00036 | Clear improvement |
| Re-admission | 1055 / 87 | +3.75 | 0.883 | No improvement |
| In-hospital mortality | 1056 / 95 | −1.66 | 0.059 | Borderline, not robust |
| 30-day mortality | 1054 / 123 | −1.82 | 0.055 | Borderline, not robust |
| Surgery needed | 1055 / 31 | +0.69 | 0.191 | No improvement; few events |
| Length of hospital stay | 1052 | −4.73 | 0.013 | Moderate improvement |
| Total blood transfusions | 1056 | +3.93 | 0.964 | No improvement |

**Supplementary table 4**: Exploratory assessment of non-linearity for age using restricted cubic splines. ICU – intensive care unit; AIC – Akaike information criterion.
