# Supplementary material for: Age-related differences in the presentation, management, and outcomes of lower gastrointestinal bleeding: a retrospective multinational cohort study
Source: Lancet Reg Health Eur. 2026 Jul 9;68:101775. doi: 10.1016/j.lanepe.2026.101775 (PMC13380016; doi:10.1016/j.lanepe.2026.101775)
Supplement: Supplementary Table S5 [file mmc5.docx]

| **Variables** | **Missing** |
| --- | --- |
| Age | 0/1058 (0%) |
| Sex | 0/1058 (0%) |
| Charlson comorbidity index | 0/1058 (0%) |
| Oakland score | 33/1058 (3.1%) |
| ABC score | 177/1058 (16.7%) |
| ALIBI score | 29/1058 (2.7%) |
| Haemostatic endoscopic therapy | 9/1058 (0.9%) |
| Binary variables | <1.2% |

**Supplementary table 5**: Missing data for variables included in multivariable models.
