# Supplementary material for: Age-related differences in the presentation, management, and outcomes of lower gastrointestinal bleeding: a retrospective multinational cohort study
Source: Lancet Reg Health Eur. 2026 Jul 9;68:101775. doi: 10.1016/j.lanepe.2026.101775 (PMC13380016; doi:10.1016/j.lanepe.2026.101775)
Supplement: Supplementary Table S6 [file mmc6.docx]

| **Outcome** | **Younger reference interval*** | **60-64 years** | **≥65 years** | **Holm-significant pairwise contrasts** | **Interpretation** |
| --- | --- | --- | --- | --- | --- |
| Red blood cell transfusion count | 0.965 | 1.159 | 1.364 | 60-64 vs younger; ≥65 vs younger | 60-64 closer to younger than to ≥65 |
| Haemostatic endoscopic therapy | 0.185 | 0.255 | 0.203 | 60-64 vs younger; ≥65 vs 60-64 | Non-linear pattern with a peak near the cutoff |
| Surgery in the first 30 days | 0.037 | 0.043 | 0.027 | None | No clear age-related separation |
| Hospital admission | 0.639 | 0.766 | 0.842 | All pairwise contrasts | Gradual/intermediate increase around the cutoff |
| Length of stay | 6.84 | 9.11 | 10.65 | All pairwise contrasts | Gradual/intermediate increase around the cutoff |
| ICU admission | 0.094 | 0.105 | 0.044 | ≥65 vs younger; ≥65 vs 60-64 | 60-64 closer to younger interval; lower ICU admission in the oldest stratum |
| Readmission | 0.060 | 0.067 | 0.090 | None | 60-64 closer to younger interval |
| In-hospital mortality | 0.051 | 0.067 | 0.102 | None | No clear separation after correction |
| 30-day mortality | 0.047 | 0.070 | 0.139 | ≥65 vs younger; ≥65 vs 60-64 | 60-64 closer to younger interval |

**Supplementary table 6**: Sex-standardised marginal outcome estimates across age intervals close to the 65-year cutoff. Values represent predicted probabilities for binary outcomes and predicted means for count outcomes. Holm correction was applied within each outcome. *The younger reference interval refers to the non-overlapping age interval below 60 years used in the exploratory analysis. ICU – intensive care unit.
