# Supplementary material for: Age-related differences in the presentation, management, and outcomes of lower gastrointestinal bleeding: a retrospective multinational cohort study
Source: Lancet Reg Health Eur. 2026 Jul 9;68:101775. doi: 10.1016/j.lanepe.2026.101775 (PMC13380016; doi:10.1016/j.lanepe.2026.101775)
Supplement: Supplementary Table S7 [file mmc7.docx]

| **Variables** | **Events/**  **observed N** | **Effect measure for ≥65 vs <65 years (95% CI)** | **Country random-effect SD** | **Centre random-effect SD** | **Interpretation** |
| --- | --- | --- | --- | --- | --- |
| Red blood cell transfusion (any) | 477/1057 | OR 2.23 (1.60-3.11) | 0.96 | 0.07 | Higher in older patients |
| Endoscopy performed | 899/1058 | OR 0.67 (0.39-1.14) | 1.53 | 0.06 | No clear separation after clustering |
| Haemostatic endoscopic therapy | 205/1049 | OR 1.06 (0.72-1.57) | 0.65 | 0.07 | No clear separation after clustering |
| Computed tomography angiography | 245/1058 | OR 1.10 (0.76-1.59) | 0.84 | 0.07 | No clear separation after clustering |
| Embolization | 7/1055 | OR 0.32 (0.09-1.18) | 0.19 | 0.06 | Sparse events; interpret cautiously |
| Surgery in the first 30 days | 31/1056 | OR 0.92 (0.42-2.05) | 0.27 | 0.07 | No clear separation after clustering |
| Hospital admission | 838/1058 | OR 2.54 (1.77-3.64) | 1.40 | 0.07 | Higher in older patients |
| Length of stay | 1052 | IRR 1.45 (1.37-1.53) | 0.38 | 0.18 | Longer in older patients |
| Total blood transfusions | 1056 | IRR 1.17 (1.01-1.35) | 0.46 | 0.11 | Higher count in older patients |
| ICU admission | 60/1045 | OR 0.54 (0.30-0.96) | 0.98 | 0.07 | Lower in older patients |
| Rebleeding | 137/1055 | OR 1.36 (0.83-2.25) | 0.75 | 0.06 | Directionally higher; interval crosses null |
| Readmission | 87/1055 | OR 1.19 (0.66-2.11) | 0.42 | 0.07 | No clear separation after clustering |
| In-hospital mortality | 95/1056 | OR 1.91 (1.04-3.50) | 0.44 | 0.07 | Higher in older patients |
| 30-day mortality | 124/1054 | OR 2.83 (1.54-5.22) | 0.36 | 0.07 | Higher in older patients |

**Supplementary table 7**: Exploratory centre/country cluster-adjusted age-group sensitivity models. Models included age group (≥65 versus <65 years) and sex as fixed effects, with random intercepts for country and participating centre nested within country. Binary outcomes are reported as odds ratios (ORs); count outcomes are reported as incidence rate ratios (IRRs). CI – confidence interval; ICU – intensive care unit.
