# Supplementary material for: Age-related differences in the presentation, management, and outcomes of lower gastrointestinal bleeding: a retrospective multinational cohort study
Source: Lancet Reg Health Eur. 2026 Jul 9;68:101775. doi: 10.1016/j.lanepe.2026.101775 (PMC13380016; doi:10.1016/j.lanepe.2026.101775)
Supplement: Study Protocol [file mmc8.docx]

**Study protocol**

Lower gastrointestinal bleeding differences between older and younger adults: European cohort study

**Collaborative approach**

This study will be conducted through a collaborative network of European gastroenterology centres with experience in the management of gastrointestinal bleeding. A central management team will coordinate the study and will be responsible for protocol development, study oversight, and data management.

The central management team will include Francisco Vara-Luiz, Carolina Palma, Paulo Mascarenhas, Marta Patita, and Jorge Fonseca. Participating investigators at each centre will be responsible for identifying eligible patients and collecting study data according to the predefined protocol.

The full management and authorship group will be reported in the final publication, and collaborators will be acknowledged in accordance with ICMJE authorship guidelines.

**Introduction**

Lower gastrointestinal bleeding (LGIB) is a common medical emergency associated with substantial morbidity, mortality, and healthcare resource use. In the context of progressively ageing populations across Europe, the clinical profile of patients presenting with LGIB is changing. Older adults now represent an increasing proportion of affected patients and are frequently characterised by multimorbidity, polypharmacy, and widespread use of antithrombotic therapy.

Despite advances in diagnostic and therapeutic strategies, important knowledge gaps remain regarding contemporary patient characteristics, bleeding aetiologies, management patterns, and clinical outcomes in this evolving population. In particular, few recent studies have comprehensively examined the combined influence of ageing, multimorbidity, and antithrombotic exposure on the clinical course of LGIB, although these factors increasingly define the typical patient presenting with this condition.

Better understanding of these changes is essential to inform guideline development, refine risk stratification tools, optimise healthcare resource allocation, and support clinical decision-making in increasingly complex and medically vulnerable populations. However, current guidelines provide limited age-specific recommendations and do not fully address the challenges associated with the management of LGIB in older adults.

**Hypothesis / Research question**

We hypothesise that LGIB in older adults represents a distinct clinical phenotype shaped primarily by age-related multimorbidity, polypharmacy and reduced physiological reserve, rather than by differences in diagnostic or therapeutic management.

The primary research question is whether age-related biological and clinical factors modify the presentation, underlying causes, and short-term outcomes of LGIB when comparing older and younger adults in contemporary European practice. Addressing this question may help clarify whether LGIB in ageing populations should be conceptualised not solely as a gastrointestinal event but as a manifestation of systemic clinical vulnerability.

**Methods**

Study design

This study will be a multinational, observational cohort study conducted across participating centres in Europe.

Inclusion criteria

Consecutive adult patients aged ≥18 years will be eligible if they were admitted with evidence of recent LGIB within the preceding three days during a 12-month enrolment period (January 1 to December 31, 2024). Evidence of LGIB was defined by the presence of haematochezia or melena.

Exclusion criteria

- Pregnancy.
- Insufficient clinical data
- No evidence of gastrointestinal bleeding
- Documented gastrointestinal bleeding on upper endoscopy

Data collection and management

Clinical, laboratory, diagnostic, therapeutic, and outcome data related to the index bleeding episode and subsequent clinical course up to 30 days will be collected retrospectively from electronic medical records at each participating centre using predefined study variables and standardised definitions.

Variables will include demographics, comorbidities (liver cirrhosis, congestive heart failure and diabetes), Charlson Comorbidity Index, previous LGIB, antiplatelet and anticoagulant therapy, clinical presentation (melena, haematochezia, haematochezia + melena), haemodynamic status, laboratory parameters at admission (haemoglobin, platelet count, international normalised ratio), LGIB aetiology, and endoscopic haemostatic therapy. Bleeding risk scores (Oakland, age-blood tests-comorbidities [ABC], and acute lower gastrointestinal bleeding and in-hospital mortality [ALIBI]) will be calculated as previously described.

A data dictionary specifying all study variables and definitions will be developed by the study coordinating committee and shared with all participating investigators to ensure consistency in data collection across centres.

Each participating centre will be responsible for identifying eligible patients and entering anonymised data into the study database. Local investigators will verify the accuracy and completeness of the collected data before submission.

The coordinating team will perform central data screening to identify missing values, inconsistencies, and potential data entry errors. Queries will be communicated to local investigators when clarification or verification is required.

All data will be anonymised prior to analysis in accordance with applicable data protection regulations.

Primary and secondary endpoints

The primary endpoint of this study is 30-day all-cause mortality following an episode of LGIB.

Secondary endpoints include:

1. Rehospitalisation within 30 days, defined as any unplanned hospital admission following discharge from the index LGIB hospitalisation.
2. In-hospital mortality.
3. Requirement for red blood cell transfusion during the index admission.
4. Requirement for haemostatic intervention, including endoscopic therapy, interventional radiology, or surgery.
5. Length of hospital stay (days from admission to discharge).
6. Healthcare resource use, including intensive care unit admission.

Statistical analysis

Descriptive statistics will be used to summarise patient characteristics. Continuous variables will be reported as mean (SD) or median (IQR), as appropriate, and categorical variables as frequencies and percentages.

Group comparisons will be performed using independent t tests or Mann–Whitney U tests for continuous variables and χ² or Fisher’s exact tests for categorical variables.

Multivariable regression models will be constructed to identify factors independently associated with clinical outcomes. Binary outcomes will be analysed using logistic regression models and reported as odds ratios (ORs) with 95% confidence intervals. Count outcomes will be analysed using negative binomial regression models and reported as incidence rate ratios (IRRs).

Missing predictor data will be handled using multiple imputation techniques. A two-sided p value of <0.05 will be considered statistically significant.

**Ethics statement**

The study will be conducted in accordance with the ethical principles of the Declaration of Helsinki. The study protocol will be approved by the institutional ethics committee or review board at each participating centre. Because of the retrospective observational nature of the study and the use of anonymised routinely collected data, the requirement for informed consent may be waived according to local regulatory requirements.

**Conflicts of interest**

The authors have no conflicts of interest to declare.

**Patient and public involvement**

Patients or members of the public were not involved in the design, conduct, reporting, or dissemination plans of this study.

**Study transparency**

The study protocol was finalised before data analysis and was shared with all participating centres prior to study initiation.

**Reporting standards**

The study will be reported in accordance with the Strengthening the Reporting of Observational Studies in Epidemiology (STROBE) guidelines.

**Appendix A: Study definitions**

The following predefined clinical definitions will be applied across all participating centres to ensure consistency in data collection and interpretation.

**Lower gastrointestinal bleeding (LGIB)**

Lower gastrointestinal bleeding (LGIB) will be defined as bleeding originating distal to the ileocecal valve presenting as haematochezia and/or melena. Bleeding originating between the ligament of Treitz and the ileocecal valve will be considered mid-gastrointestinal bleeding and will be excluded if identified.

**Recent LGIB**

Recent LGIB will be defined as evidence of lower gastrointestinal bleeding occurring within the preceding three days prior to hospital presentation.

**Haematochezia**

Haematochezia will be defined as the passage of fresh or maroon-coloured blood per rectum.

**Melaena**

Melaena will be defined as black, tarry stools resulting from gastrointestinal bleeding. Patients with melaena will first undergo upper gastrointestinal endoscopy according with international guidelines.

**Haemodynamic instability**

Haemodynamic instability was defined using the first available vital signs at emergency department presentation or hospital admission:

- systolic blood pressure <90 mmHg
- heart rate >100 beats per minute
- requirement for vasopressor support

**Rebleeding**

Rebleeding will be defined as any of the following occurring after the initial bleeding episode:

- recurrence of overt lower gastrointestinal bleeding
- development of new hemodynamic instability
- a decrease in haemoglobin ≥2 g/dL after initial stabilisation

**Red blood cell transfusion**

Red blood cell transfusion will be defined as the administration of packed red blood cell units during the index hospitalisation. Red blood cell transfusion decisions should be guided by haemoglobin level, haemodynamic status, ongoing bleeding, cardiovascular comorbidity, and overall clinical condition according to guideline recommendations.

**Endoscopic haemostatic therapy**

Endoscopic haemostatic therapy will include any therapeutic endoscopic intervention performed to control active or recent bleeding, including mechanical methods (e.g., clips), thermal coagulation (e.g., argon plasma coagulation), injection therapy, or topical haemostatic agents.

**Interventional radiology**

Interventional radiology will be defined as angiographic embolisation performed to control gastrointestinal bleeding.

**Surgical intervention**

Surgery will be defined as operative intervention performed to control refractory LGIB when endoscopic and/or radiological therapies are unsuccessful or not feasible.

**Readmission**

Readmission will be defined as any all-cause unplanned hospital admission occurring within 30 days after discharge from the index LGIB hospitalisation, including but not limited to recurrent LGIB. Planned admissions, elective procedures, and scheduled follow-up admissions will not be considered readmissions.

**Intensive care unit (ICU) admission**

ICU admission will be defined as admission to an intensive care unit for monitoring or management of LGIB or related complications during the index hospitalisation.

**Thirty-day mortality**

Thirty-day mortality will be defined as death from any cause occurring within 30 days following the index LGIB episode.

**Cause of death**

Causes of death will be classified as bleeding-related when death was directly attributed to uncontrolled or recurrent gastrointestinal bleeding, haemorrhagic shock, or complications of bleeding management. Deaths classified as non-bleeding-related will include comorbidity decompensation, infection, cardiovascular events, respiratory failure, malignancy progression, or other causes not directly attributed to ongoing bleeding.

**Appendix B: Summary of data fields (case report form)**

| **Variable name** | **Coding / Units** |
| --- | --- |
| Patient_ID | Site-specific anonymised code |
| Centre | Predefined centre code |
| Age | Years |
| Age_group | <65 years / ≥65 years |
| Sex | Male / Female |
| Charlson_Comorbidity_Index | Score |
| Liver_cirrhosis | Yes / No |
| Congestive_heart_failure | Yes / No |
| Diabetes_mellitus | Yes / No |
| Previous_LGIB | Yes / No |
| Antiplatelet_therapy | Yes / No |
| Aspirin_use | Yes / No |
| Clopidogrel_use | Yes / No |
| Anticoagulant_therapy | Yes / No |
| DOAC_use | Yes / No |
| VKA_use | Yes / No |
| LMWH_use | Yes / No |
| Bleeding_presentation | Haematochezia / Melena / Haematochezia + melena |
| Haemodynamic_instability | Yes / No |
| Haemoglobin | g/dL |
| Platelet_count | x10⁹/L |
| INR | Numeric |
| Oakland_score | Score |
| ABC_score | Score |
| ALIBI_score | Score |
| LGIB_cause | Angiodysplasia / Anorectal disease / Diverticulosis / Inflammatory bowel disease / Infectious colitis / Undetermined colitis / Ischaemic proctocolitis / Radiation proctocolitis / Colorectal polyps / Postpolypectomy bleeding / Colorectal cancer / Other / Inconclusive |
| Endoscopy_performed | Yes / No |
| Endoscopy_type | Colonoscopy / Rectosigmoidoscopy |
| Time_to_endoscopy | Days |
| Therapeutic_endoscopy | Yes / No |
| Second_look_endoscopy | Yes / No |
| RBC_transfusion | Yes / No |
| Number_RBC_units | Units |
| Interventional_radiology | Yes / No |
| Surgery | Yes / No |
| Hospital_admission | Yes / No |
| ICU_admission | Yes / No |
| Length_of_stay | Days |
| Rebleeding | Yes / No |
| Rehospitalisation | Yes / No |
| In_hospital_mortality | Yes / No |
| Mortality_30_days | Yes / No |
| Cause_of_death | Bleeding-related / Non-bleeding-related |

**References**

1. Feldman M, Friedman LS and Brandt LJ (eds). Sleisenger and Fordtran’s gastrointestinal and liver disease. 11th ed. Philadelphia, PA: Elsevier, 2020.
2. Oakland K, Kahan BC, Jairath V. Diagnosis and management of acute lower gastrointestinal bleeding: guidelines from the British Society of Gastroenterology. Gut. 2019;68(5):776–789.
3. Balvardi S, Galante D. Lower Gastrointestinal Bleeding. Surg Clin North Am. 2026 Feb;106(1):133-141. doi: 10.1016/j.suc.2025.08.010.
4. Hreinsson JP, Kalaitzakis E, Gudmundsson S, Björnsson ES. Acute lower gastrointestinal bleeding: incidence, etiology, and outcomes in a population-based setting. Eur J Gastroenterol Hepatol. 2013;25(1):37–43.
5. Hreinsson JP, Gumundsson S, Kalaitzakis E. Acute lower gastrointestinal bleeding: a population-based five-year follow-up study. UEG Journal. 2019;7(8):1125–1133.
6. Vora P, Shah A, Ostaszkiewicz G, Ruigómez A, Rodríguez LAG. Thirty-year incidence and mortality trends in upper and lower gastrointestinal bleeding in the United Kingdom and Denmark. JAMA Netw Open. 2020;3(7):e2018507.
7. Uhlig K, Leff B, Kent D, Dy S, Brunnhuber K, Burgers JS, Greenfield S, Guyatt G, High K, Leipzig R, Mulrow C, Schmader K, Schunemann H, Walter LC, Woodcock J, Boyd CM. A framework for crafting clinical practice guidelines that are relevant to the care and management of people with multimorbidity. J Gen Intern Med. 2014 Apr;29(4):670-9. doi: 10.1007/s11606-013-2659-y.
8. Salive ME. Multimorbidity in older adults. Epidemiol Rev. 2013;35(1):75–83.
9. Kate V, Sureshkumar S, Gurushankari B, Kalayarasan R. Acute Upper Non-variceal and Lower Gastrointestinal Bleeding. J Gastrointest Surg. 2022 Apr;26(4):932-949. doi: 10.1007/s11605-022-05258-4.
10. Yuhara H, Corley DA, Nakahara F, Nakajima T, Koike J, Igarashi M, Suauki T, Mine T. Aspirin and non-aspirin NSAIDs increase risk of colonic diverticular bleeding: a systematic review and meta-analysis. J Gastroenterol. 2014 Jun;49(6):992-1000. doi: 10.1007/s00535-013-0905-z.
11. Dobesh PP, Fanikos J. Direct oral anticoagulants for the prevention of stroke in atrial fibrillation: bleeding risk and monitoring. J Thromb Thrombolysis. 2015;39(3):395–402.
12. Holster IL, Valkhoff VE, Kuipers EJ, Tjwa ET. New oral anticoagulants increase risk for gastrointestinal bleeding: a systematic review and meta-analysis. Gastroenterology. 2013;145(1):105–112.e15.
13. Bénard F, Barkun AN, Martel M. Recent advances in colonoscopy for lower gastrointestinal bleeding. Curr Opin Gastroenterol. 2019;35(1):33–40.
14. Radaelli F, Frazzoni L, Repici A, Rondonotti E, Mussetto A, Feletti V, Spada C, Manes G, Segato S, Grassi E, Musso A, Di Giulio E, Coluccio C, Manno M, De Nucci G, Festa V, Di Leo A, Marini M, Ferraris L, Feliziani M, Amato A, Soriani P, Del Bono C, Paggi S, Hassan C, Fuccio L. Clinical management and patient outcomes of acute lower gastrointestinal bleeding. A multicenter, prospective, cohort study. Dig Liver Dis. 2021 Sep;53(9):1141-1147. doi: 10.1016/j.dld.2021.01.002.
15. Triantafyllou K, Gkolfakis P, Gralnek IM, Oakland K, Manes G, Radaelli F, Awadie H, Camus Duboc M, Christodoulou D, Fedorov E, Guy RJ, Hollenbach M, Ibrahim M, Neeman Z, Regge D, Rodriguez de Santiago E, Tham TC, Thelin-Schmidt P, van Hooft JE. Diagnosis and management of acute lower gastrointestinal bleeding: European Society of Gastrointestinal Endoscopy (ESGE) Guideline. Endoscopy. 2021 Aug;53(8):850-868. doi: 10.1055/a-1496-8969. Epub 2021 Jun 1. Erratum in: Endoscopy. 2021 Aug;53(8):C10. doi: 10.1055/a-1528-2092.
16. Sengupta N, Feuerstein JD, Jairath V, Shergill AK, Strate LL, Wong RJ, Wan D. Management of Patients With Acute Lower Gastrointestinal Bleeding: An Updated ACG Guideline. Am J Gastroenterol. 2023 Feb 1;118(2):208-231. doi: 10.14309/ajg.0000000000002130.
17. Oakland K, Jairath V, Uberoi R, Guy R, Ayaru L, Mortensen N, Murphy MF, Collins GS. Derivation and validation of a novel risk score for safe discharge after acute lower gastrointestinal bleeding: a modelling study. Lancet Gastroenterol Hepatol. 2017 Sep;2(9):635-643. doi: 10.1016/S2468-1253(17)30150-4.
18. Laursen SB, Oakland K, Laine L, Bieber V, Marmo R, Redondo-Cerezo E, Dalton HR, Ngu J, Schultz M, Soncini M, Gralnek I, Jairath V, Murray IA, Stanley AJ. ABC score: a new risk score that accurately predicts mortality in acute upper and lower gastrointestinal bleeding: an international multicentre study. Gut. 2021 Apr;70(4):707-716. doi: 10.1136/gutjnl-2019-320002.
19. Oakland K, Guy R, Uberoi R, Hogg R, Mortensen N, Murphy MF, Jairath V; UK Lower GI Bleeding Collaborative. Acute lower GI bleeding in the UK: patient characteristics, interventions and outcomes in the first nationwide audit. Gut. 2018 Apr;67(4):654-662. doi: 10.1136/gutjnl-2016-313428.
20. Marengoni A, Angleman S, Melis R, Mangialasche F, Karp A, Garmen A, Meinow B, Fratiglioni L. Aging with multimorbidity: a systematic review of the literature. Ageing Res Rev. 2011 Sep;10(4):430-9. doi: 10.1016/j.arr.2011.03.003.
21. Lanas Á, Carrera-Lasfuentes P, Arguedas Y, García S, Bujanda L, Calvet X, Ponce J, Perez-Aísa Á, Castro M, Muñoz M, Sostres C, García-Rodríguez LA. Risk of upper and lower gastrointestinal bleeding in patients taking nonsteroidal anti-inflammatory drugs, antiplatelet agents, or anticoagulants. Clin Gastroenterol Hepatol. 2015 May;13(5):906-12.e2. doi: 10.1016/j.cgh.2014.11.007.
22. Abraham NS, Hartman C, Richardson P, Castillo D, Street RL Jr, Naik AD. Risk of lower and upper gastrointestinal bleeding, transfusions, and hospitalizations with complex antithrombotic therapy in elderly patients. Circulation. 2013 Oct 22;128(17):1869-77. doi: 10.1161/CIRCULATIONAHA.113.004747.
23. Prasitvarakul K, Attanath N, Chang A. Comparison of scoring systems for predicting clinical outcomes of acute lower gastrointestinal bleeding: A prospective cohort study. World J Surg. 2024 Feb;48(2):474-483. doi: 10.1002/wjs.12053.
24. Dajti E, Frazzoni L, Castellet-Farrús S, Guardiola J, Sinagra E, Anderloni A, Ferrara F, Gkolfakis P, Camus Duboc M, Mandarino FV, Sadeghi A, Lorenzo-Zúñiga V, Perez S, Triantafyllou K, Curado MP, Facciorusso A, Collatuzzo G, Hassan C, Radaelli F, Fuccio L; ALIBI Study Group. In-hospital mortality in patients with lower gastrointestinal bleeding: development and validation of a prediction score. Endoscopy. 2025 Aug;57(8):839-848. doi: 10.1055/a-2541-2312.
25. Brandt LJ, Feuerstadt P, Longstreth GF, Boley SJ; American College of Gastroenterology. ACG clinical guideline: epidemiology, risk factors, patterns of presentation, diagnosis, and management of colon ischemia (CI). Am J Gastroenterol. 2015 Jan;110(1):18-44; quiz 45. doi: 10.1038/ajg.2014.395.
26. Calderwood AH, Tosteson TD, Walter LC, Hua P, Onega T. Colonoscopy utilization and outcomes in older adults: Data from the New Hampshire Colonoscopy Registry. J Am Geriatr Soc. 2022 Mar;70(3):801-811. doi: 10.1111/jgs.17560.
27. Brunker LB, Boncyk CS, Rengel KF, Hughes CG. Elderly Patients and Management in Intensive Care Units (ICU): Clinical Challenges. Clin Interv Aging. 2023 Jan 22;18:93-112. doi: 10.2147/CIA.S365968.
28. Pilotto A, Custodero C, Crudele L, Morganti W, Veronese N, Franceschi M. Age-related changes of the gastrointestinal tract. Lancet Gastroenterol Hepatol. 2026 Jan;11(1):59-70. doi: 10.1016/S2468-1253(25)00235-3.
29. Ferreira-Gonzalez S, Matsumoto T, Hara E, Forbes SJ. Senescence, Aging and Disease Throughout the Gastrointestinal System. Gastroenterology. 2025 Dec;169(7):1357-1379. doi: 10.1053/j.gastro.2025.06.010.
30. Zhang C, Huang Q, Liu X, Wang J, Wang J, Song J, Song R, Su H, Mei Q. Frailty and risk of gastrointestinal bleeding: a prospective cohort study based on UK biobank. Front Public Health. 2025 Jul 4;13:1625869. doi: 10.3389/fpubh.2025.1625869.
